# Functional Improvement and Regression of Medial Hypertrophy in the Remodeled Pulmonary Artery after Correction of Systemic Left-to-Right Shunt

**DOI:** 10.1038/srep37684

**Published:** 2016-11-25

**Authors:** Chih-Hsin Hsu, Jun-Neng Roan, Jyh-Hong Chen, Chen-Fuh Lam

**Affiliations:** 1 Institute of Clinical Medicine, National Cheng Kung University Hospital, College of Medicine, National Cheng Kung University, 138 Sheng-Li Road, Tainan 704, Taiwan; 2 Department of Internal Medicine, National Cheng Kung University Hospital, College of Medicine, National Cheng Kung University, 138 Sheng-Li Road, Tainan 704, Taiwan; 3 Division of Cardiovascular Surgery Department of Surgery, National Cheng Kung University Hospital, College of Medicine, National Cheng Kung University, 138 Sheng-Li Road, Tainan 704, Taiwan; 4 Department of Internal Medicine, China Medical University, 95 Xueshi Road, Taichung City 40454, Taiwan; 5 Department of Anesthesiology, Buddhist Tzu-Chi General Hospital and Tzu-Chi University School of Medicine, 707 Zhongyang Road Section 3, Hualien City 970, Taiwan; 6 Department of Anesthesiology, Taipei Medical University Hospital and School of Medicine, College of Medicine, Taipei Medical University, 252 Wu-Xing Street, Taipei 110, Taiwan

## Abstract

The presence of systemic left-to-right shunt and increased pulmonary blood flow can result in right heart failure and pulmonary arteriopathy. Correction of left-to-right shunt has been shown to improve cardiac function and physical performance. However, the cardiopulmonary remodeling processes following cessation of left-to-right shunt have yet to be reported. In this experimental study, excessive pulmonary flow was restored through ligation of the aortocaval fistula in rats with flow-induced pulmonary hypertension. The cardiopulmonary morphometric functions were assessed, and phenotypic switching of pulmonary vascular smooth muscle cells (VSMC) was determined. Ligation of aortocaval fistula significantly attenuated pulmonary blood flow and right ventricular mass, and potentiated the isometric contraction of pulmonary artery. Inflammatory cytokines IL-1β and IL-6 were reduced in the lung after ligation. Reduction of pulmonary blood flow restored the expressions of smooth muscle myosin heavy chain and α-smooth muscle actin in pulmonary artery, indicating the switching of VSMCs to the contractile phenotype. Our study demonstrated that normalization of pulmonary blood flow in flow-induced pulmonary hypertension reverses the remodeling in the right ventricle and pulmonary artery. The remodeling process of flow-induced pulmonary hypertension is functionally and morphometrically reversible by inducing transdifferentiation of pulmonary VSMC to contractile phenotypes and modulation of tissue inflammatory cytokines.

Pulmonary arteriopathy is a unique vascular abnormality, which may develop in patients with pulmonary hypertension secondary to increased pulmonary blood flow, and can be observed in conditions such as left-to-right shunt congenital heart defects and systemic arteriovenous shunt^1^,^2^. The characteristic vascular changes of pulmonary arteriopathy include intimal hyperplasia/fibrosis, medial hypertrophy, extensive extracellular matrix modulation and in more severe cases, the formation of plexiform lesions^1^,^3^. These changes lead to decreased compliance of pulmonary vasculature and changes in vasoreactivity^4^,^5^. Patients with flow-induced pulmonary hypertension have significantly higher perioperative mortality and a lower long-term survival rate after cardiac operation^5^,^6^,^7^. After reviewing 63 patients with truncus arteriosus who underwent surgical repair, Hanley *et al*. concluded that presence of pulmonary hypertension is associated with worse clinical outcomes^6^. The presence of pulmonary hypertension in patients with congenital heart disease before cardiac surgery is also a strong predictor for postoperative in-hospital death^8^. The overall survival rateof end-stage renal disease patients who receive hemodialysis via arteriovenous fistulas is significantly reduced with the coexistence of flow-induced pulmonary hypertension^9^.

Congenital heart diseases, intimal fibrosis and medial proliferation were found to regress over time (years) following a banding procedure of the pulmonary artery in patients with pulmonary hypertension secondary to left-to-right shunt, indicating that increased pulmonary blood flow is one of the fundamental pathophysiologies of flow-induced pulmonary hypertension^10^. In hemodialysis patients who eventually received renal transplants and closure of arteriovenous fistula, pulmonary artery pressure was found to decline significantly from 49.8 to 38.6 mmHg^11^. Our clinical studies also demonstrated that closure of atrial septal defect (ASD) significantly reduced pulmonary artery pressure and serum levels of B-type natriuretic peptide (BNP), improving the cardiac performance and physical performance in these patients^12^. Although flow-induced pulmonary artery is most likely clinically reversible following cessation of the excessive pulmonary blood flow^8^, the remodeling process of pulmonary circulation and cardiac morphology has not been previously characterized. Thus, the aim of this study was to characterize the morphometric function, vasoreactivity, and vascular smooth muscle phenotypic modulation of the pulmonary artery and right heart following cessation of excessive pulmonary blood flow in a rat model of prolonged systemic left-to-right shunt.

## Results

### Ligation of high-flow aortocaval fistula improved right ventricular function

**Figure 1:**
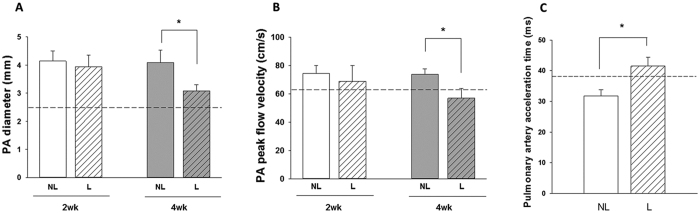
Measurement of cardiopulmonary morphometric function using the transthoracic echocardiography. Assessment of pulmonary artery (PA) diameter (**A**), PA pressure (**B**) and PA acceleration time (PAAT) measured at 4 weeks after fistula ligation (**C**). The peak flow velocity across pulmonary valve was significantly reduced at 4 weeks after aortocaval fistula ligation, and the diameter of PA was simultaneously reduced in corresponding to decrease in PA pressure. On the contrary, the PAAT increased significantly after ligation of aortocaval fistula, suggesting the reduction of pulmonary vascular resistance. Dotted lines indicate the levels of sham animals. Results were analyzed using unpaired t-test, and data are presented as mean ± SD. n = 6–8 different animals in each group. NL: non-ligation group; L: ligation group.

**Figure 2:**
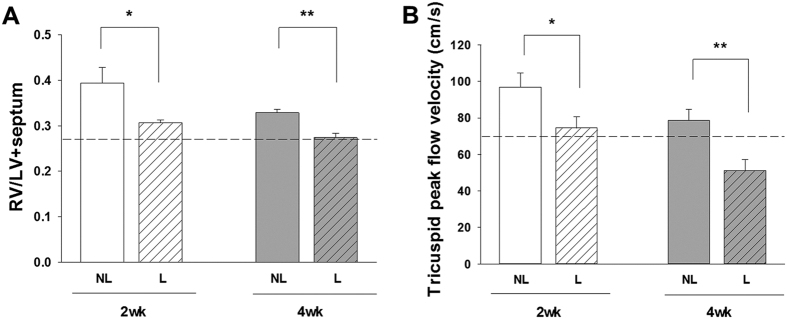
Measurement of cardiopulmonary morphometric function using the transthoracic echocardiography and RV/LV + S mass ratio. Assessment of right ventricular (RV) hypertrophy (**A**) and RV pressure (**B**). Ligation of aortocaval fistula at 2 and 4 weeks significantly reduced the tricuspid peak flow velocity, and the simultaneously attenuated the right ventricular hypertrophy measured by the RV/LV + S mass ratio. Dotted lines indicate the levels of sham animals. Results were analyzed using unpaired t-test, and data are presented as mean ± SD. n = 6–8 different animals in each group. *P < 0.05 compared with no ligation.

**Figure 3:**
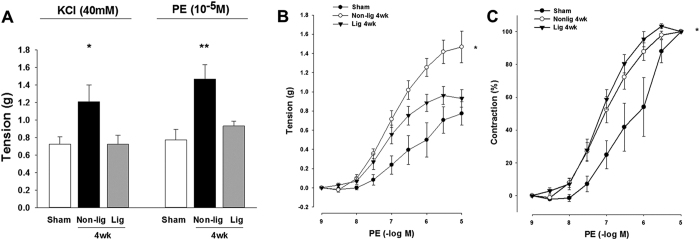
The overall cardiopulmonary changes related to the left ventricular (LV) function were assessed by the LV ejection fraction (**A**) and degree of pulmonary congestion (lung-wet-to-dry ratio, LWDR; (**B**). Results were analyzed using unpaired t-test, and data are presented as mean ± SD. n = 6–8 different animals in each group. *P < 0.05 compared with no ligation.

The diameter and blood flow rate in the main pulmonary artery as measured by echocardiography *in vivo* indicated that pulmonary artery diameter and peak flow velocity were significantly reduced at 4 weeks after ligation (Fig. 1A and B). The pulmonary vascular resistance assessed by the pulmonary artery acceleration time (PAAT) was restored at 4 weeks after ligation of fistula (Fig. 1C). Reduction of excessive pulmonary blood flow also significantly attenuated the tricuspid peak flow velocity and ratio of ratio of right ventricle (RV) weight to left ventricle plus septum (LV + S) weight (RV/LV + S) mass ratio at 2 weeks and 4 weeks after ligation of aortocaval fistula (Fig. 2A and B). However, ligation of aortocaval fistula did not affect the ejection fraction of the left ventricle and lung wet-to-dry ratio (LWDR) (Fig. 3A and B). Furthermore, the systolic blood pressures measured in the descending aorta did not differ between rats with patent and ligated aortocaval fistula (84 ± 10.3 vs 81 ± 9.5 mmHg, respectively; P = 0.50, n = 4–5 different animals in each group).

### Reduction of pulmonary blood flow potentiated pulmonary arterial vasomotor function

**Figure 4:**
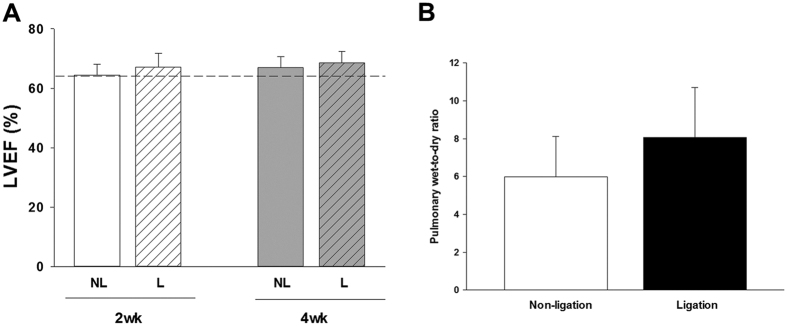
Measurements of isometric force of pulmonary artery in the sham-operated, and rats with flow-induced pulmonary hypertension. (**A**) Contraction responses of pulmonary artery to KCl (40 mM) and maximal concentration of phenylephrine (PE, 10^−5^ M). (**B**) Contraction responses of pulmonary artery to cumulative addition of phenylephrine (PE, 10^−9^ to 10^−5^ M). (**C**) Sensitivity of pulmonary contraction response to phenylephrine shown by % contraction. Results were analyzed using two-way RM ANOVA, and data are presented as mean ± SD. n = 6 different animals in each group. *P < 0.05 compared with AV fistula ligation.

The contractions of the isolated pulmonary artery in response to KCl depolarization and α1-adrenergic stimulation were significantly enhanced in rats with flow-induced pulmonary hypertension (Fig. 4A and B). Reduction of the excessive pulmonary blood flow following ligation of aortocaval fistula significantly decreased the pulmonary artery contractility response (Fig. 4A and B), but the sensitivity of the remodeled pulmonary artery to phenylephrine stimulation remained increased at 4 weeks after aortocaval fistula ligation (Fig. 4C).

### Changes in tissue inflammatory cytokines after reduction of pulmonary blood flow

**Figure 5:**
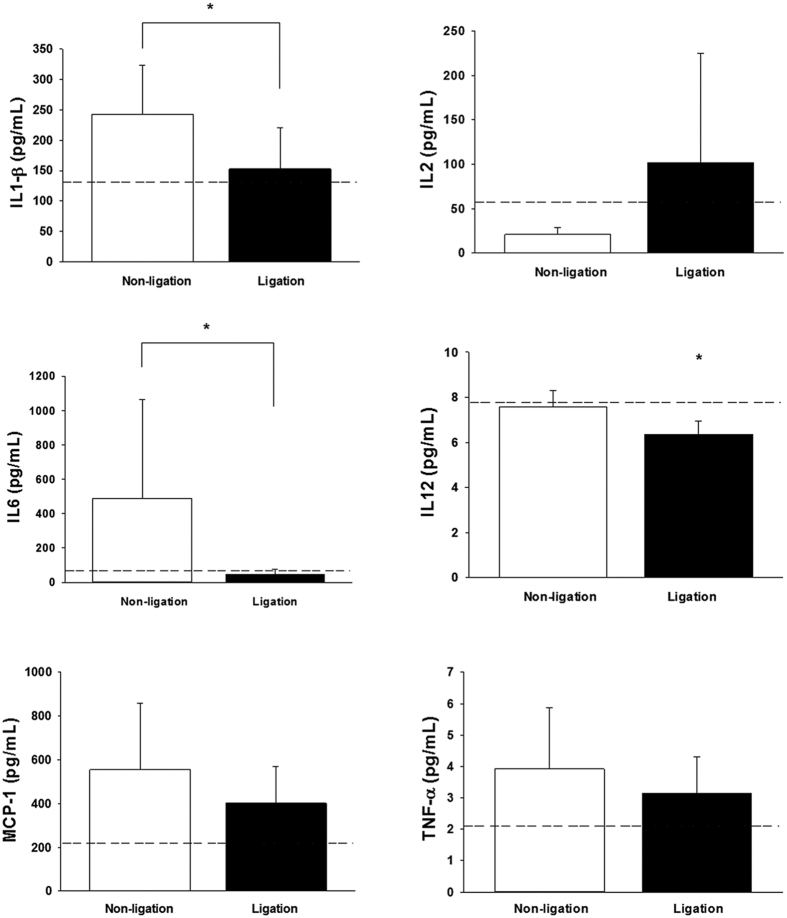
Measurement of tissue concentrations of pro-inflammatory cytokines (IL-1β, IL-2, IL-6, IL-12, MCP-1 and TNF-α) in lung homogenates using the ELISA assay. *P < 0.05 compared with non-ligation group. n = 6–8 different animals in each group. Data were were analyzed using the Mann-Whitney U test and are shown as mean ± SD. Dotted lines denote the levels of sham-operated animals.

Compared with rats with persistent high pulmonary flow, rats that received aortocaval fistula ligation displayed significantly suppressed tissue concentrations of IL-1β, IL-6 and IL-12 in the lung homogenates, while levels of IL-2, MCP-1, and TNF-α did not differ (Fig. 5).

### Phenotypic presentatio of vascular smooth muscle cells (VSMC) after reduction of pulmonary blood flow

**Figure 6:**
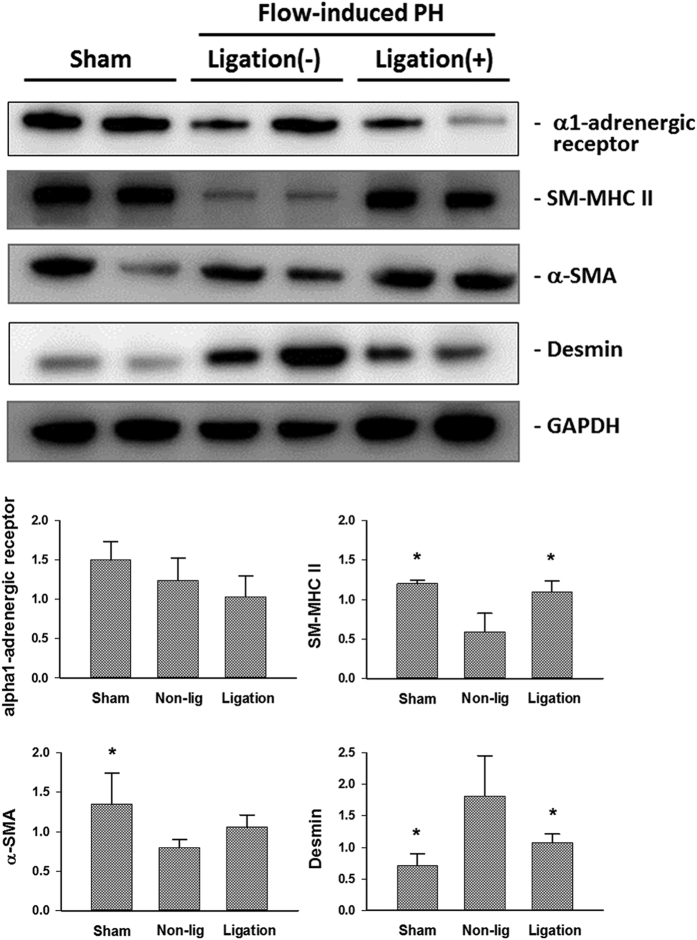
Representative blots of protein expressions of smooth muscle myosin heavy chain (SM-MHC)-II, α-smooth muscle actin (SMA), desmin and matrix metalloproteases (MMP)-2 in the isolated pulmonary artery of experimental rats. Ligation of aortocaval fistula restored the synthetic to contractile phenotyping of vascular smooth muscle cells by enhanced expressions of SM-MHC-II and α-SMA, and suppressed expression of desmin. *P < 0.05 compared with non-ligation group. n = 6 different animals in each group. Each band represents an independent tissue sample obtained from a different animal and the bands were visualized using an automated imaging system (UPV BioSpectrum) under the same experimental conditions. Data were analyzed by one-way ANOVA and are shown as mean ± SD.

In the pulmonary artery exposed to consistent high blood flow, the expressions of smooth muscle myosin heavy chain (SM-MHC)-II and α-smooth muscle actin (SMA) were down-regulated, whereas the expression of desmin was up-regulated (Fig. 6). Following the cessation of excessive pulmonary flow, the protein levels of the phenotypic markers of contractile VSMC (enhanced SM-MHC-II and reduced desmin) were restored (Fig. 6). The expressions of α1-adrenergic receptor in the pulmonary artery homogenates were similar despite the changes in pulmonary blood flow (Fig. 6).

### Regression of pulmonary arterial medial hypertrophy after reduction of pulmonary blood flow

**Figure 7:**
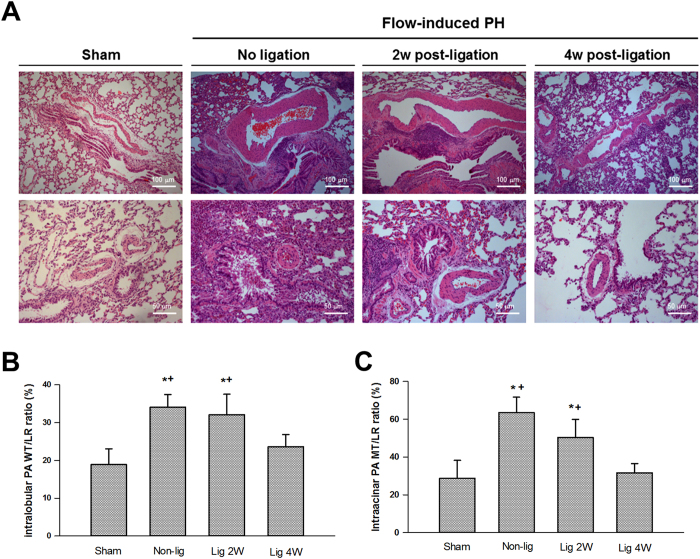
Representative lung sections of animals with and without flow-induced pulmonary hypertension (**A**). Morphometric analysis of the medial thickness of the intralobular (ϕ 500–1000 μm, (**B**) and intraacinar (ϕ <250 μm, **C**) pulmonary artery. Thickness of medial layer is expressed as wall thickness to lumen radius (WT/LR) ratio (%). Thickness of medial layer was significantly reduced 4 weeks (lig 4w) after ligation of aortocaval fistula. *P < 0.05 compared with sham and ^†^P < 0.05 compared with lig 4w. Magnifications are 100× for the upper panel and 200× for the lower panel. Analysis was performed in 5–6 different lung sections in each group, and the medical thickness was measured in at least 1 intralobular and 2 intraacinar pulmonary artery in each tissue section using the ImageJ software. Data were analyzed by one-way ANOVA and are shown as mean ± SD.

Hypertrophy of the medial layer in the intralobular and intraacinar pulmonary arteries developed after prolonged increase in pulmonary blood flow (Fig. 7). Medial thickness was progressively reduced in the remodeled pulmonary artery after ligation of aortocaval fistula, and returned to the baseline levels at 4 weeks after reduction in pulmonary flow (Fig. 7).

## Discussion

Consistent with the clinical presentation in patients with flow-induced pulmonary hypertension who eventually received ASD occlusion, the experimental findings of our animal study support the clinical evidence that changes in the reduction of pulmonary blood flow following application of ASD occluder improved the cardiopulmonary physiology and functional performance of these patients. This animal study further characterized the cardiopulmonary remodeling process, vasoreactivity, and pulmonary vascular smooth muscle phenotypic switching after cessation of excessive pulmonary blood flow.

In the pulmonary artery exposed to increased blood flow following the creation of aortocaval fistula in rats, the contractions to suboptimal KCl and cumulative phenylephrine stimulation were significantly increased in the pulmonary artery, and the EC_50_ of phenylephrine was reduced, suggesting that flow-induced pulmonary hypertension results in a serial morphological and molecular changes in the vascular medial layer. Previous studies reported that an increase in the catecholamines-mediated trophic effect and activation of Rho-kinase could be the mechanisms responsible for flow-induced vascular smooth muscle hypertrophy, proliferation, and increased reactivity to vasoconstrictors^9^,^10^. Occlusion of aortocaval fistula that reduced left-to-right shunt flow significantly attenuated the contractility of isolated pulmonary artery to KCl depolarization and α1-adrenergic stimulation that were comparable to sham-operated animals (consider rephrasing). However, the sensitivity response of the pulmonary artery to phenylephrine remained significantly elevated (lower EC50 of PE) in the animals that received aortocaval ligation. These vascular reactivity changes illustrated that reduction in pulmonary blood flow restored the medial hypertrophy of pulmonary artery, but the sensitivity of adrenergic receptors to α1-agonism in the remodeled pulmonary artery might require a prolonged period of time to reestablish. This study failed to demonstrate a significant difference in the expression of α1-adrenergic receptors in the isolated pulmonary arteries, which suggest that these vasomotor reactions were mainly derived from the functional changes in the medial layer of pulmonary artery.

At basal conditions, VSMC predominantly present as the contractile phenotype. Buuns *et al*. showed that high flow rates enhanced proliferation of VSMC and reduced mRNA levels of desmin and α-SMA in the rat mesenteric artery, indicating the de-differentiation of VSMCs to synthetic phenotype in response to shear stress^11^. In the de-differentiated state, VSMCs reenter the cell cycle and increase their rate of proliferation, migration, and synthesis of extracellular matrix components^13^. α-SMA and SM-MHC II are considered contractility-related genes and decreased expression of these genes have been recognized as markers for de-differentiation of VSMC^11^,^14^,^15^. In line with these findings, the pulmonary artery obtained from rats with flow-induced pulmonary hypertension showed reduced expressions of α-SMA and SM-MHC-II, suggesting VSMC de-differentiates from the contractile phenotype to the synthetic phenotype in response to increased blood flow after creation of the aortocaval fistula. De-differentiation of VSMCs is essential for vascular remodeling and enlargement of the internal diameter of blood vessels in order to normalize the increased shear stress^11^. After the aortoacaval fistula was ligated and the normal pulmonary blood flow was restored, VSMC undertook differentiation and switched from the synthetic phenotype (increased flow) to the contractile phenotype (basal flow), evidenced by the enhancement of protein expressions of SM-MHC II and down-regulation of desmin in the pulmonary artery.

This study also measured the regulation of inflammatory cytokines in the lung to characterize the remodeling process after cessation of increased pulmonary blood flow. The most important inflammatory cytokines associated with primary or secondary pulmonary hypertension are IL-1β and IL-6^16^. Elevated tissue levels of IL-1β were found in PAH patients and correlate with a worse outcome^17^. In small clinical trials, treatment with the IL-1β receptor antagonist improved clinical symptoms of patients with secondary pulmonary hypertension^17^. IL-6 is another inflammatory cytokine that has been shown to involve significantly in the pathogenesis of pulmonary artery hypertension and hypoxia-induced pulmonary hypertension^16^. Serum levels of IL-6 were significantly higher in patients with pulmonary hypertension as compared with normal controls^18^. The IL-6 levels were also well correlating with survival of the pulmonary hypertensive patients and provided superior predictive value for the functional improvement in these patients than the traditional clinical tests (the 6-minute walking distance and hemodynamic measurements)^19^. In this study, concentrations of IL-1β and IL-6 were significantly reduced in the lung tissues of rats receiving aortocaval fistula ligation, suggesting the improvement in the pulmonary physiology and remodeling after cessation of excessive pulmonary blood flow. Consistent with similar levels of lung water content (lung wet-to-dry ratio), the absence of changes in IL-2, MCP-1 and TNF-α levels might simply illustrate that inflammatory process and tissue edematous reaction was not remarkable.

This study looked into the performance of heart and pulmonary circulation after reduction of pulmonary blood flow. Transthoracic echocardiographic examination demonstrated that the peak flow velocities across tricuspid and pulmonary valves were both enhanced at 4 weeks after aortocaval fistula ligation. Tricuspid and pulmonary peak flow velocities are useful sonographic parameters for assessing the pressures in the right ventricle and pulmonary artery, respectively. Pulmonary peak flow velocity was significantly reduced at 2 and 4 weeks after aortocaval fistula ligation and tricuspid peak flow velocity was reduced at 4 weeks after ligation. In addition, pulmonary artery acceleration time was significantly increased following aortocaval ligation, indicating that the increased pulmonary vascular resistance in rats with flow-induced pulmonary hypertension (reduced PAAT) was normalized after cessation of left-to-right shunt^20^. These hemodynamic changes suggested that the pressure and resistance gradients across these circulating systems (right atrium to right ventricle and right ventricle to pulmonary artery) were reduced after reduction of pulmonary blood flow. Since pulmonary arterial circulation is a high compliance system with high-flow and low-pressure circuit^21^, the reduction in the pulmonary artery pressure thus led to the advanced alteration in the right ventricle. As expected with the reduction in right ventricular and pulmonary pressures, the RV/LV + S mass ratio was decreased in rats receiving aortocaval fistula ligation, suggesting improvement in right ventricular hypertrophy. With decreased pulmonary artery pressure and improvement in right ventricular size, the histological sections of the remodeled pulmonary artery were studied to characterize the morphological changes after normalization of pulmonary blood flow. The thickness of the medial layers in the conduit (intralobular, outer diameter of 500–1000μm) and more resistant (intraacinar, outer diameter of <250μm) pulmonary arteries was significantly reduced at 4 weeks after ligation. Particularly, the thickness of medial layer regressed to the normal levels after 4 weeks. Collectively, these hemodynamic and histological changes highlighted that the unloading of hemodynamic stress in the pulmonary artery leads to reversal of pulmonary arteriopathy and right heart remodeling. Normalization of pulmonary blood flow not only restores the phenotypic presentation of VSMC, but also the pathomorphometry of pulmonary artery. Abe *et al*. recently tested the effects of blood flow on the disease progress of pulmonary arteriopathy in a rat model of Sugen/hypoxia-induced pulmonary hypertension^22^. Their results reported that the development of occlusive neointimal lesion and regional inflammatory response was significantly attenuated in rats with reduced blood flow by pulmonary artery banding. Thus, our study is supported by the concept that hemodynamic stress is prerequisite to the development and progress of pulmonary hypertension^22^.

There are a number of limitations in this study. First, we assessed the pulmonary hemodynamics by the tricuspid peak flow velocity and PAAT, and determined the right ventricular remodeling by the RV/LV + S ratio. In clinical practice, measurement of tricuspid annular plane systolic excursion (TAPSE) and right ventricular volumetry is useful echocardiographical methods for quantification of right ventricular function. However, we found that TAPSE and right ventricular ejection fraction were more likely subjected to technical bias during measurement in small animals. Therefore, TAPSE and right ventricular EF were not reported in this study. Second, the molecular mechanisms for pulmonary artery remodeling and VSMC phenotypic switching secondary to blood flow changes were not determined by pharmacological approaches. Since modulation of the potential signaling molecules, such as cyclooxygenase-2 or Rho kinase, significantly affects vascular remodeling and blood flow in the aortocaval fistula^23^, the pharmacological effects on pulmonary hemodynamics and lung histomorphometry are not confined to pulmonary circulation, which make the interpretation of drug effect on flow-induced pulmonary hypertension unfeasible. Third, constant increased pulmonary blood flow will eventually result in congestive heart failure which activate the endogenous neurohormonal system. Activations of the renin angiotensin aldosterone system, cathecolamines, endothelin and natriuretic peptides are known to modulate the remodeling of cardiovascular system in response to volume or pressure overload^24^. However, our previous study showed that clinically significant heart failure developed 8 weeks after creation of aortocaval fistula in rats^25^. In the absence of hypertension and signs of heart failure (evidenced by the echocardiographic findings and lung water content), the neurohormonal system was less likely being activated in this study.

In conclusion, this experimental model of aortocaval fistula ligation which mimicked occlusion of left-to-right shunt ASD demonstrated that normalization of pulmonary blood flow in subjects with flow-induced pulmonary hypertension reverses the remodeling in the right ventricle and pulmonary arterial circulation, and potentiates the vascular reactivity of pulmonary artery. The remodeling process of flow-induced pulmonary hypertension is most likely reversible and these changes are closely related to differentiation and switching of VSMC in the pulmonary artery and modulation of tissue inflammatory cytokines.

## Methods

### Rat model of flow-induced pulmonary hypertension

All animal experimental procedures were approved by the Institutional Animal Care and Use Committee (IACUC; The National Cheng Kung University, Tainan, Taiwan) and were performed in accordance with the Guide and Use of Laboratory Animals (Institute of Laboratory Animal Resources). Sprague-Dawley rats (weight 200–250 g) were anesthetized with inhaled isoflurane (2–3% v/v in oxygen). Following a midline abdominal incision, the inferior vena cava (IVC) and aorta were exposed. Vascular clamps were placed across the aorta and IVC just above the aortic bifurcation and below the origin of renal vessels. The aorta was punctured with a 20 G disposable needle at the level below renal vessels. The needle was gradually introduced across the aorta and penetrated the neighboring wall of IVC. The needle was then withdrawn and the puncturing point was closed by purse-string suturing. Vascular clamps were removed and abdominal wall was closed in layers. The sham-operated rats underwent laparotomy, cross-clamping of the aorta and IVC for 30 seconds without puncturing, and the placement of purse-string suturing at the lumbar aorta.

### Cessation of flow-induced pulmonary hypertension

Six weeks after creation of aortocaval fistula when flow-induced pulmonary hypertension is known to establish^5^, the animals were re-anesthetized and the fistula was identified and ligated to interrupt the excessive pulmonary blood flow.

### Transthoracic echocardiography and hemodynamic measurements

Echocardiography was performed in anesthetized rats using a high-resolution *in vivo* imaging system (Sonos S500, M24214A Ultrasound System) equipped with a 12 MHz probe (Agilent Technologies). The animals were placed in supine position during examination. The cardiac dimensions and function were assessed using the two-dimensional (long- and short-axis) and motion-mode (M-mode) images. The transducer was maximally aligned to optimize endocardial visualization and spectral displays of Doppler profiles. M-mode measurements were performed according to recommendation of the American Society of Echocardiography. M-mode measurement of left ventricular wall thickness and cavity size were performed in the parasternal short-axis view. Right ventricular end-diastolic dimension was measured in the parasternal short-axis view just below the level or aortic valve. Pulse-wave Doppler of pulmonary outflow was recorded in parasternal view at the level of aortic valve. The sample volume was placed proximal to the pulmonary leaflets and aligned to maximize laminar flow. The PAAT (in milliseconds) was measured from the start to of peak velocity in the pulmonary artery flow. The tricuspid valve was interrogated for the present of tricuspid regurgitation with color and continuous-wave Doppler in the apical four chamber view. The transducer was aligned to achieve the maximal peak velocity on tricuspid regurgitation. All measurements were performed by an experienced sonographer who was blinded to the treatment group. To measure the systemic blood pressure, a needle connected with a pressure transducer was inserted directly into the descending aorta following laparotomy, and the measurements were displayed by a computerized data acquisition system.

### Right ventricular hypertrophy measurement

Immediately after euthanasia, the hearts were removed and dissected to isolate the free wall of the right ventricle from the left ventricle and septum. The ratio RV/LV + S was used as an index of right ventricular hypertrophy.

### Analysis of isolated pulmonary artery vasoreactivity

Vascular rings (2 mm in length) were isolated from the right and left pulmonary arteries. Vascular rings were mounted in organ chambers containing 25 ml of Krebs solution containing (in mmol/L) 118.6 NaCl, 4.7 KCl, 2.5 CaCl_2_, 1.2 MgSO_4_, 1.2 KH_2_PO_4_, 25.1 NaHCO_3_, 10.1 glucose, and 0.026 EDTA. The chambers were maintained at 37 °C and aerated continuously with 94% O_2_/6% CO_2_. Changes in isometric force were recorded using an isometric force-displacement transducer (Grass FT03; Grass Instrument). Each ring was gradually stretched to 1.5 g. After a 45-min equilibration period, the rings were contracted by an addition of KCl (40 mM) and increasing concentrations of phenylephrine (PE, 10^−9^ to 10^−5^ M). Papaverine (3 × 10^−4^ M) was used to induce complete relaxation of the vessels. All experiments were performed in vessels with intact endothelium.

### Lung-wet-to dry ratio (LWDR)

The intermediate lobe of the right lung was excised and weighed immediately. Lung tissues were dried in an oven at 80 °C for 12 h and reweighed. The LWDRs were obtained by dividing the mass of the initial specimen by the mass of the dried specimen.

### Western blot

Soluble protein extracts (50 μg) were loaded into polyacrylamide gels (9–12%) and transferred onto the nitrocellulose membranes. Membranes were immunoblotted overnight with mouse monoclonal anti-α-SMA, anti-desmin, and anti- SM-MHC-II antibodies. After washing, the membranes were incubated with horseradish peroxidase-linked secondary antibodies for 1 hour at room temperature. Chemiluminescence enhanced bands were visualized using an automated imaging system (UVP Biospectrum). Protein levels were quantified by scanning densitometry (ImageJ Software; 1.48 v, National Institutes of Health, USA).

### ELISA assay for cytokines

The tissue levels of pro-inflammatory cytokines (IL-1β, IL-6, IL-10, MCP-1 and TNFα) in the lung homogenates were determined using rat cytokine ELISA assay kits (Signosis Inc.), according to the manufacturer’s instructions. Spectrophotometrical changes following addition of detection reagents were measured using an ELISA-plate reader.

### Histological examination

Lung tissues were immersed in 10% formaldehyde for 24 hours. Paraffin-embedded tissues were sectioned and stained with Hematoxylin and Eosin, and Verhoeff’s stains. Thickness of medial layer in the intralobular (ϕ 500–1000 μm) and intraacinar (ϕ < 250 μm in diameter) pulmonary arteries was measured under a light microscope (100–200X). The medial thickness is expressed as thickness of medial layer (WT, in micrometers) to the lumen radius (LR, lumen radius was computed from the area of pulmonary arterioles in micrometers) (WT/LR) ratio^26^. Area of medial wall was measured using the ImageJ Software (1.48 v, National Institutes of Health, USA).

### Statistical analysis

When experiments were performed in parallel on tissues from different groups of animals and the normality assumptions were met, unpaired t-test or analysis of variance (ANOVA) were employed; otherwise the Mann-Whitney U test or Kruskal-Wallis test was used, as appropriate. Multiple comparisons were performed using Bonferroni correction. A P value less than 0.05 was considered to be statistically different in all tests. All the statistical analyses were performed using the SigmaStat Scientific Software (version 2.0; Jandel Corporation, San Rafael, CA).

## Additional Information

**How to cite this article**: Hsu, C.-H. *et al*. Functional Improvement and Regression of Medial Hypertrophy in the Remodeled Pulmonary Artery after Correction of Systemic Left-to-Right Shunt. *Sci. Rep*. **6**, 37684; doi: 10.1038/srep37684 (2016).

**Publisher's note:** Springer Nature remains neutral with regard to jurisdictional claims in published maps and institutional affiliations.
